# Rhabdomyolysis and Acute Renal Dysfunction as Initial Manifestations of Monoclonal Gammopathy of Renal Significance

**DOI:** 10.7759/cureus.34759

**Published:** 2023-02-08

**Authors:** Prathyusha Gudapati, Anmar Al- Sultani, Ashish Parmar, Reena Motwani, Peter Fortkort

**Affiliations:** 1 Internal Medicine, UNC Health Southeastern, Lumberton, USA; 2 Internal Medicine, Campbell University School of Osteopathic Medicine, Buis Creek, USA

**Keywords:** extra renal manifestations of monoclonal gammopathy of renal significance, monoclonal immunoglobulin deposition disease, multiple myeloma, light chain deposition disease, rhabdomyolysis, monoclonal gammopathy of renal significance, monoclonal gammopathy

## Abstract

Monoclonal gammopathy of renal significance (MGRS) is a rare heterogeneous group of kidney disorders that encompasses all disorders caused by deposition of monoclonal protein (M-protein) and its light or heavy chain fragments secreted by pre-malignant or non-malignant B-cell clones in patients who do not meet the diagnostic criteria for multiple myeloma (MM) or other B-cell malignancies. MGRS can manifest as glomerular diseases, tubulopathies, or vascular involvement with varying clinical presentations, making the diagnosis of MGRS challenging. In patients with high clinical suspicion based on preliminary blood and urine studies, the evaluation of MGRS begins with a renal biopsy followed by monoclonal studies and cytogenetic analysis. There is no standard treatment protocol for MGRS, and the current consensus suggests a clone-directed approach. If not identified and treated early, MGRS often results in poor outcomes and can lead to extrarenal manifestations, such as cardiogenic shock. Herein, we present a case involving a 43-year-old male with a rare presentation of rhabdomyolysis, rapidly progressing renal dysfunction, and cardiac dysfunction. A bone marrow biopsy did not meet the diagnostic criteria for MM or other B-cell malignancies, while a renal biopsy revealed Kappa light chain cast nephropathy, which led to the final diagnosis of MGRS.

## Introduction

Plasma cell dyscrasias, also known as monoclonal gammopathies (MGs) or paraproteinemias, are a heterogeneous group of immunosecretory disorders of the hematologic system. Plasma cell dyscrasias are monoclonal proliferations of lymphoplasmacytic cells in the bone marrow that produce a monoclonal immunoglobulin or paraprotein and, occasionally, the deposition of these paraproteins in tissues [1]. Clinical manifestations of MGs range from completely asymptomatic to end-organ damage without meeting the criteria for overt multiple myeloma or other malignant lymphoproliferative neoplasms (MPNs) [2]. The incidence of these MGs is two to three times higher in African Americans, and men are more frequently affected than females [1]. A circulating M-protein is present in 3% of people aged 50 years and older, which increases to approximately 5% after the age of 70 [2,3]. If this circulating M-protein is below 30 g/L and monoclonal bone marrow plasma cells are less than 10%, a diagnosis of monoclonal gammopathy of undetermined significance (MGUS) is made in the absence of MM-defining events, such as hypercalcemia, renal impairment, anemia, or lytic bone lesions [2,3]. The kidneys are often involved, and various kidney diseases in the absence of hematological malignancies such as MM, Waldenstrom macroglobulinemia (WM), and chronic lymphocytic leukemia (CLL) are increasingly recognized [4]. Since monoclonal gammopathy plays a direct role in significant renal damage in these disorders, the International Kidney and Monoclonal Gammopathy Research Group proposed the term “monoclonal gammopathy of renal significance" (MGRS) to discriminate these rare disorders from benign MGUS [4].

MGRS manifests in a continuously evolving list of clinical presentations [4,5]. MGRS is prevalent in 0.32% and 0.53% of people ages greater than 50 and 70 years, respectively [6]. The risk of progression within the first year of diagnosis of overt MM is significantly higher in MGRS patients (10%) compared to MGUS patients (1%) [7]. Because of the wide spectrum of manifestations of MGRS, diagnosis can often be challenging, and it is difficult to establish a pathogenic link between the M-protein and renal dysfunction [2]. MGRS also confers an increased risk of progression to end-stage renal disease (ESRD). Therefore, early recognition and treatment are critical due to the possibility of halting the progression of MGRS and, in some cases, reversal of renal dysfunction [2]. However, chemotherapy regimens for MGRS are poorly defined, and current evidence favors clone-directed therapy [2,8].

Light chain deposition as an isolated MG or in the context of MM or WM most commonly occurs in the kidney due to progressive accumulation from plasma filtration, as the renal plasma flow accounts for 20% of total plasma flow [9]. Approximately 45% of these patients with kidney involvement have rapidly progressive renal failure eventually progressing to ESRD, thus requiring hemodialysis [10]. Extra renal light chain deposition, although rare, can occur in virtually any tissue, with the heart and liver being the most commonly affected organs [9,11,12]. Cardiac manifestations of arrhythmias, conduction disturbances, and congestive heart failure are reported in as many as 80% of cases of light chain deposition disease (LCDD) [9,12]. Though rhabdomyolysis due to light chain deposition in the cellular membrane of muscle fibers associated with Kappa light chain multiple myeloma (Kappa LCMM) was previously reported, rhabdomyolysis associated with MGRS has yet to be reported [13,14]. A literature review was conducted using PubMed, Cochrane, Scopus, and Embase, and no prior reports of rhabdomyolysis in MGRS were found. In this paper, we report the first case of rhabdomyolysis associated with MGRS.

## Case presentation

A 43-year-old African American male with a past medical history of hypertension was presented to our hospital with a one-day history of slurred speech, confusion, and left-sided weakness. The presenting vitals were as follows: temperature of 98.7 °Fahrenheit, heart rate (HR) of 148 beats per minute (bpm), respiratory rate (RR) of 19 breaths per minute (BPM) and blood pressure (BP) of 138/103. Shortly after his arrival, the patient developed respiratory distress with an RR of 65 and an oxygen saturation of 73%. He was placed on high-flow oxygen, which was transitioned to bilevel positive airway pressure (BiPAP). Arterial blood gas on BiPAP showed a pH of 7.25, partial pressure of carbon dioxide (pCO_2_) of 32, partial pressure of oxygen (pO_2_) of 245 and bicarbonate level of 15.3. Initial labs were not suggestive of anemia (hemoglobin 15.1g/dL), or hypercalcemia (calcium 8.2 mg/dL). However, they were consistent with acute renal failure with a serum creatinine level of 7.9 mg/dL, high anion gap metabolic acidosis with an anion gap of 24, and serum bicarbonate level of 15 mmol/L. His creatinine on outpatient labs 13 months prior to presentation was 1.1 mg/dL. He also had hyperammonemia with an ammonia level of 108 ug/dL, elevated brain natriuretic peptide (BNP) level of 1,262 pg/mL and acute transaminitis with aspartate aminotransferase (AST) of 516 IU/L and alanine aminotransferase (ALT) of 211 IU/L. His AST and ALT levels 13 months prior were 15 IU/L and 11 IU/L respectively. A urinalysis showed 3+ blood with no red blood cells and 3+ protein. Rhabdomyolysis was also noted, with creatine kinase (CK) levels trending up from 9,000 units/L to a peak of 18,000 units/L. At the time, he was not on any nephrotoxic medications, such as renin angiotensin inhibitors. Computed tomography (CT) head without contrast did not show any acute intracranial abnormalities. Chest CT without contrast demonstrated mild cardiomegaly, significant lower lobe pneumonia, small foci of pneumonitis within the right middle lobe, and pre-carinal lymphadenopathy.

The patient was eventually intubated due to altered mentation and was admitted to the intensive care unit (ICU). He went into cardiogenic shock on day two and required pressor support. Workup for acute metabolic encephalopathy was performed, including testing for neurological, endocrine, rheumatological, and infectious causes, and was found negative. His extensive workup included a meningitis panel, cerebrospinal fluid (CSF) cultures with venereal disease research laboratory test (VDRL), blood cultures, urine cultures, respiratory cultures, Lyme workup, Borrelia workup, Rickettsia workup, HIV workup, bacterial antigen panel, antineutrophil cytoplasmic antibodies (ANCA) panel, glomerular basement membrane antibodies, rheumatoid factor, Sjogren’s antibodies, antinuclear antibody with reflex, and Smith/nuclear ribonucleoprotein (RNP) antibodies. An abdominal ultrasound and renal ultrasound performed on day two demonstrated a normal liver, borderline splenomegaly, and normal kidneys. A 2D echocardiogram performed on day three showed a severely reduced ejection fraction (EF) estimated at 20-25%. Initially, acute renal failure was attributed to rhabdomyolysis and possible hypoperfusion from cardiogenic shock. The patient’s presentation was thought to be the result of multiple coexisting processes.

The patient’s renal function progressively further declined, and he was initiated on hemodialysis within the first week of ICU admission for refractory metabolic acidosis (an anion gap of 22 and bicarbonate of 15), progressive uremic symptoms with creatinine of 11.1, and anuria. The acute nature of his presentation and the relatively insufficient medical history made narrowing down the patient’s diagnosis rather difficult. Due to multiorgan involvement, primary amyloidosis (AL amyloidosis) was high on our differential. An investigation later revealed that the patient had overt proteinuria with a urine protein of 905 mg/dL and a protein-to-creatinine ratio of 5.963 in a spot urine protein test obtained after five dialysis sessions. His beta 2 microglobulin was elevated to 17.19 mg/L (normal 0.8-2.34). A serum protein electrophoresis and immunofixation electrophoresis (SPEP/IFE) test was performed, which did not detect the presence of a monoclonal spike (M-spike) in the sample (Table 1). However, the serum free light chain (SFLC) assay showed Kappa free light chains (FLC) levels elevated to 1,435.8 mg/L and Lambda FLC levels elevated to 113 mg/L, with a Kappa/Lambda ratio of 12.71 (Table 2).

**Table 1:** Protein Electrophoresis, Serum

Protein Fraction	Result g/dl	Reference Range
Albumin	2.66	3.5-5.60 g/dl
Albumin 1 serum	0.39	0.10-0.20 g/dl
Albumin 2 serum	1.00	0.40-0.90 g/dl
Beta Serum	0.96	0.50-1.20 g/dl
Gamma Globulin Serum	0.79	0.50-1.30 g/dl

**Table 2:** Serum Free Light Chains

	Ref Range & Units	Values
Ig Kappa Free Light Chain	3.3-19.4mg/L	1435.8
Free Lambda Lt chains, S	5.6-26.3 mg/L	113
Kappa/Lambda Ratio, S	0.26-1.65	12.71

**Figure 1:**
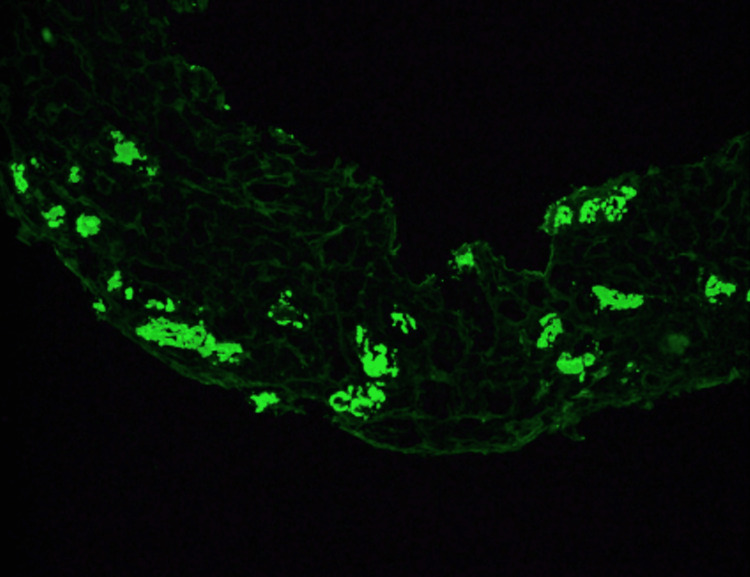
Renal Biopsy Demonstrating Kappa Positive Tubular Casts

**Figure 2:**
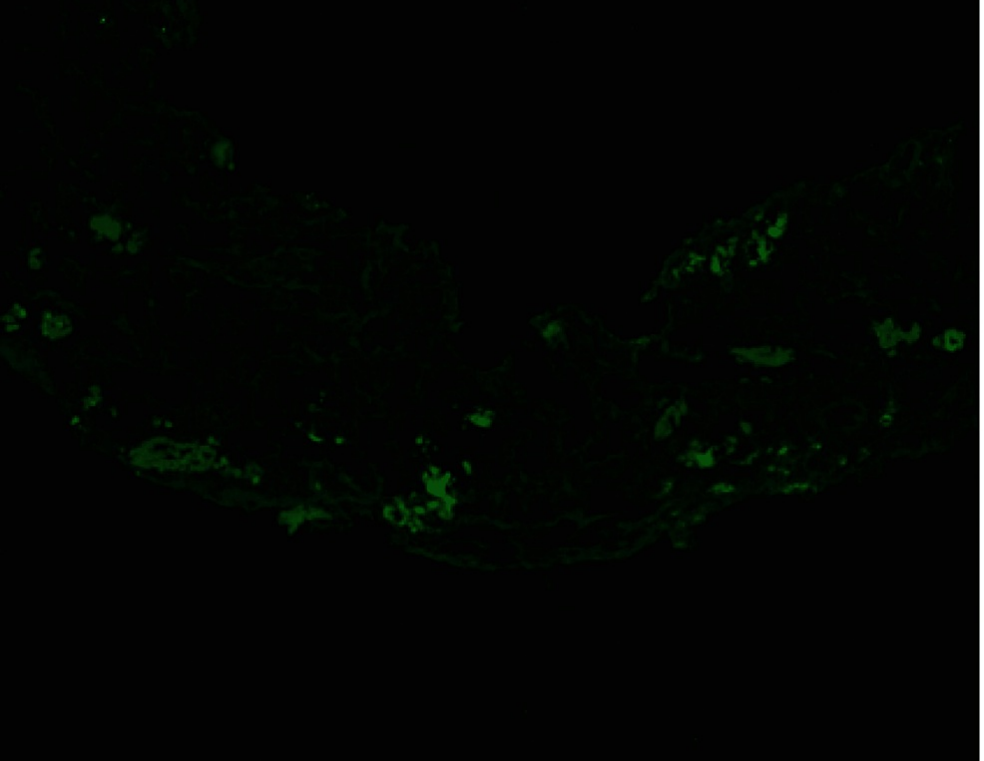
Renal Biopsy Demonstrating Lambda Negative Casts

**Figure 3:**
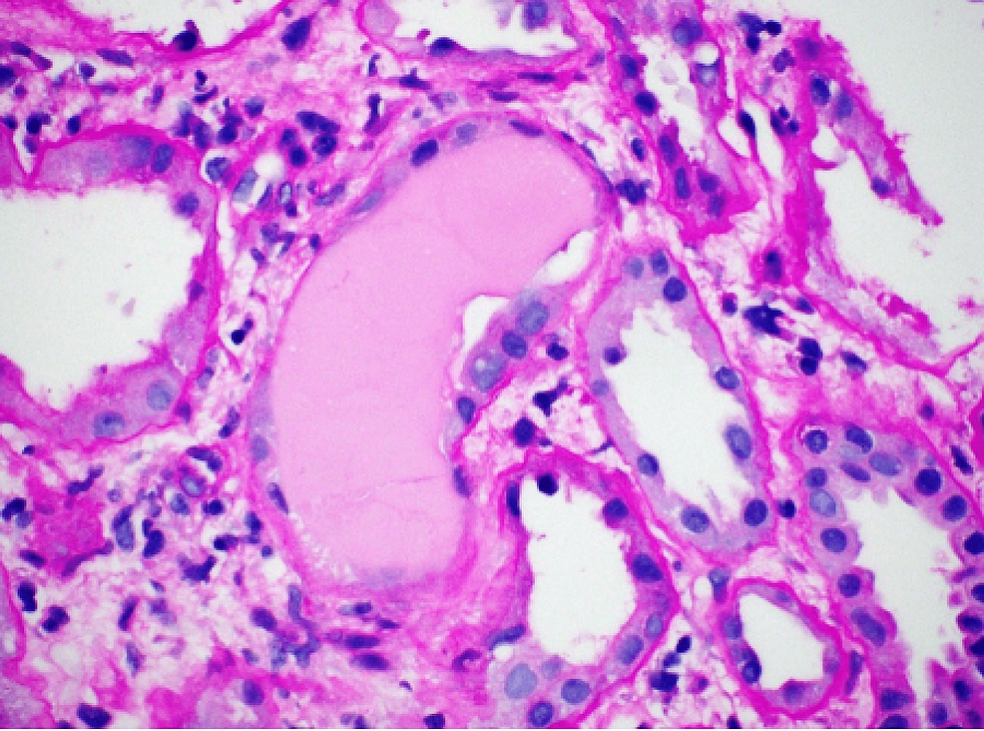
Renal Biopsy Demonstrating Periodic Acid-Schiff (PAS) Negative Tubular Casts

A renal biopsy was performed on day 16 to further investigate the cause of the renal failure. The tissue biopsies were stained with hematoxylin and eosin (H and E), Periodic Acid-Schiff (PAS), trichrome, Jones stains, antisera specific against immunoglobulin G (IgG), immunoglobulin A (IgA), immunoglobulin M (IgM), complement component 3 (C3), complement component 1q (C1q), albumin, fibrinogen, Kappa and Lambda light chains. The biopsy showed evidence of Kappa light chain cast nephropathy (Figure 1). Biopsy also demonstrated that the casts were Lambda negative (Figure 2). Congo red staining was also performed and negative for amyloid deposits (Figure 3). Leukocytic infiltrates, segmental sclerosis, necrosis, and crescents were not evident on the biopsy. Moderate interstitial fibrosis with tubular atrophy, edema, and patchy mononuclear cell interstitial cell infiltrates was also noted. Non-atrophic tubules demonstrated features of acute injury, such as epithelial attenuation, denudation of tubular basement membranes, intraluminal debris, and apical cytoplasmic blebbing. There were also occasional foci of mild lymphocytic tubulitis. An ultrastructural study revealed patchy effacement of podocyte foot processes. Glomerular basement membranes demonstrated normal trilaminar structure and mean width and are focally wrinkled in an ischemic pattern. The patient received corticosteroids in short pulses at high doses (pulse-dose steroid therapy) while awaiting further evaluation. His overall clinical status improved except for his ESRD and heart failure with reduced ejection fraction (HFrEF). A positron emission tomography-computed tomography scan (PET CT) skull base to thigh was performed, and the patient was negative for abnormal metabolic activity. Cardiac catheterization was also performed and revealed non-ischemic cardiomyopathy.

A bone marrow biopsy showed less than 5% plasma cell clones. Thus, his oncologist determined that the patient met the requirements for diagnosis of MGRS, and the patient was started on clone-directed chemotherapy with cyclophosphamide, bortezomib, and dexamethasone (CyBorD). His regimen was eventually changed to CyBorD + daratumumab. The patient could not be started on goal-directed medical therapy for his heart failure due to his low blood pressure. He received a life vest due to his EF of 20-25%, with a plan to re-evaluate for an implantable cardioverter defibrillator (ICD) six months after initiation of chemotherapy. The patient’s symptoms ultimately resolved, with the exception of ESRD. The most recent SFLC assay showed mildly elevated Kappa FLC levels at 3.45 mg/L, normal Lambda FLC levels, and mildly elevated Kappa/Lambda ratio of 2.14. His EF recovered to >55% (calculated as 53%) within 12 months of therapy initiation, and there was no evidence of hematological progression. The patient is currently awaiting autologous stem cell transplant (ASCT).

## Discussion

MGRS is a rare group of kidney disorders encompassing all disorders caused by M-proteins in patients who do not meet the diagnostic criteria for MM or other B-cell malignancies [2,5,15]. The clinical presentations of MGRS manifest as various combinations of proteinuria, hematuria, acute or chronic renal insufficiency, hypocomplementemia, and hypertension [5,15]. In patients presenting with progressively declining kidney function, atypical clinical course of chronic kidney disease, microscopic hematuria, proteinuria and/or proximal tubular dysfunction, it is important to distinguish MGRS from kidney disorders unrelated to monoclonal gammopathy [5,15]. In patients with high clinical suspicion of having MGRS based on preliminary blood and urine studies, the evaluation for MGRS begins with a renal biopsy, which can then be followed by monoclonal studies and cytogenetic analyses if an MGRS-associated lesion is identified [4]. Finally, MGRS workup should also include a complete skeletal survey [8].

The type of MGRS-associated renal lesion is determined by the structural and physicochemical properties of the M-protein [4]. M-proteins can either have a direct toxic effect on kidneys or indirect autoantibody-mediated complement factor deposition in kidneys [15,16]. MGRS-associated renal lesions are categorized based on the presence or absence of monoclonal immunoglobulin deposits in the kidney [4,8]. These deposits can be further subcategorized into organized deposits and non-organized deposits based on the ultrastructure of the deposits [4,8]. Organized MGRS renal lesions include immunoglobulin-related amyloidosis, immunoglobulin-related glomerulonephritis, monoclonal fibrillary glomerulonephritis, cryoglobulinemic glomerulonephritis, crystal globulin glomerulonephritis, crystal storing histiocytosis, and light chain proximal tubulopathy [4,8]. Non-organized MGRS renal lesions include C3 glomerulopathy, thrombotic microangiopathy, monoclonal immunoglobulin deposition disease (MIDD), and proliferative monoclonal glomerulonephritis (PGNMID) [4,8].

Currently, there are no well-defined treatments for MGRS. The most effective evidence-based treatment strategy is clone-directed therapy based on combinations of various chemotherapeutic agents in patients with severe renal disease. For instance, in the case of IgG-, IgA-, or light chain-associated MGRS, bortezomib-based anti-myeloma therapy directed against plasma cell clones to preserve kidney function is used [2,8]. In IgM-clone MGRS, rituximab is combined with either dexamethasone and cyclophosphamide or bendamustine [2,8]. The goal of MGRS treatment is to preserve kidney function, achieve a durable hematological response, and prevent recurrence after renal transplantation rather than the prolongation of life [8,17]. However, in patients with advanced renal disease and extra-renal/cardiac involvement, as is the case with our patient, the goal of treatment is to preserve extrarenal organs. In such patients, CyBorD demonstrated good responses in preliminary trials [8]. Furthermore, ASCT in conjunction with clone-directed therapy has been shown to prolong remission in patients eligible for ASCT [2,8,17]. Patients with MGRS who also have hypertension and/or proteinuria should be treated with renin-angiotensin system inhibitors unless they have AL amyloidosis [2,8]. Renin-angiotensin inhibitors should be considered only in hypertensive AL amyloidosis patients, as these patients are prone to orthostatic hypotension [8].

Although rare, reports have shown that the deposition of monoclonal light chains, predominantly Kappa light chains, leads to amyloid myopathies, and muscle biopsies earlier in the course of disease are often non-diagnostic [18]. This light chain deposition in the cellular membranes of muscle fibers was thought to induce damage to muscles, leading to the release of muscle components into blood, thus causing myoglobinuria [13,14]. It has been well-established that not all patients with rhabdomyolysis have myoglobin casts [19]. Our patient also exhibited evidence of liver injury in addition to rhabdomyolysis, renal dysfunction and cardiac dysfunction. Taken together with previous studies noting that virtually any tissue can be affected in LCDD, our patient’s rhabdomyolysis could be due to light chain muscle deposition. Our patient did not receive pulse-dose steroids until after the biopsy results and had already progressed to ESRD. His overall clinical status and labs improved after pulse-dose steroid therapy, except for renal dysfunction and cardiac dysfunction. Although pulse-dose steroid therapy alone has been used as induction therapy in young patients with MM due to excellent anti-plasma cell anti-B cell activity, pulse-dose steroid therapy in MGRS is usually part of the chemotherapy regimen [20,21]. There is no current evidence so far for a role of pulse-dose steroid therapy alone or standard protocol on how and when pulse-dose steroid therapy should be utilized during rapid progression of MGRS. Our case brings attention to the probable role of the early administration of steroids in halting the rapid progression of renal dysfunction before progression to ESRD in patients with high clinical suspicion for MGRS.

## Conclusions

Our case highlights the rapidly progressive course of MGRS and the impact aggressive management has on delaying hematological progression. MGRS should be suspected in all patients with nonmalignant or premalignant monoclonal gammopathies who present with unexplained kidney dysfunction. Early detection is crucial, and clone-directed treatment regimens improve outcomes.
